# GERD—Barrett—Adenocarcinoma: Do We Have Suitable Prognostic and Predictive Molecular Markers?

**DOI:** 10.1155/2013/643084

**Published:** 2013-03-20

**Authors:** Romana Illig, Eckhard Klieser, Tobias Kiesslich, Daniel Neureiter

**Affiliations:** ^1^Institute of Pathology, Paracelsus Medical University/Salzburger Landeskliniken (SALK), Muellner HauptstraBe 48, 5020 Salzburg, Austria; ^2^Department of Internal Medicine I, Paracelsus Medical University/Salzburger Landeskliniken (SALK), Muellner HauptstraBe 48, 5020 Salzburg, Austria

## Abstract

Due to unfavorable lifestyle habits (unhealthy diet and tobacco abuse) the incidence of gastroesophageal reflux disease (GERD) in western countries is increasing. The GERD-Barrett-Adenocarcinoma sequence currently lacks well-defined diagnostic, progressive, predictive, and prognostic biomarkers (i) providing an appropriate screening method identifying the presence of the disease, (ii) estimating the risk of evolving cancer, that is, the progression from Barrett's esophagus (BE) to esophageal adenocarcinoma (EAC), (iii) predicting the response to therapy, and (iv) indicating an overall survival—prognosis for EAC patients. Based on histomorphological findings, detailed screening and therapeutic guidelines have been elaborated, although epidemiological studies could not support the postulated increasing progression rates of GERD to BE and EAC. Additionally, proposed predictive and prognostic markers are rather heterogeneous by nature, lack substantial proofs, and currently do not allow stratification of GERD patients for progression, outcome, and therapeutic effectiveness in clinical practice. The aim of this paper is to discuss the current knowledge regarding the GERD-BE-EAC sequence mainly focusing on the disputable and ambiguous status of proposed biomarkers to identify promising and reliable markers in order to provide more detailed insights into pathophysiological mechanisms and thus to improve prognostic and predictive therapeutic approaches.

## 1. Introduction

In western countries, the particular importance of gastroesophageal reflux disease (GERD) as a main risk factor for Barrett's esophagus (BE) and esophageal adenocarcinoma (EAC) promoted by obesity, hiatus hernia, and tobacco use has increased constantly [1, 2]. Chronic injury of the gastro-esophageal junction by gastric acid or bile juice induces and promotes initially reversible metaplastic changes of the squamous epithelia which is confirmed by endoscopic examination and histomorphology [3–7]. The classical GERD-BE-EAC sequence postulates a stepwise progression over different stages of dysplasia [8, 9]. However, the postulated consecutive sequence during cancerogenesis of BE has not been proven up to now [10]. Reid et al. characterized this issue as “the paradox of Barrett's esophagus,” pointing out that the majority of EACs (95%) arise without prior diagnosis of BE or GERD which possibly indicates that steps of the proposed linear BE-EAC development are skipped. Nevertheless, consequent surveillance of patients with GERD and concomitant BE within well-defined time intervals with biopsy of suspicious lesions may prevent dysplasia—caused by epithelial injuries due to GERD—from developing into invasive cancer.

Although no increase of EAC incidence was postulated in epidemiologic studies, about 5% of patients with GERD and 0.5% with BE developed EAC [2, 11–13].

As dysplasia and adenocarcinoma are diagnosed by pathologists routineously (based on Haematoxylin-Eosin-stained biopsies), the question arises how the “risk progression” of GERD to BE and further to dysplasia and EAC can be evaluated and predicted by prognostic molecular markers and ideally may predict therapeutic success. In this paper, we try to refer to these FAQs and to provide a panel of diagnostic and predictive markers.

## 2. Definition of GERD, BE, EAC, and Types of Requested Prognostic and Predictive Markers

(i) GERD describes the chronic reflux of gastric acid or bile fluid to the esophagus resulting in metaplastic changes of the normal squamous esophageal tissue to columnar epithelium (BE) (for review, see [5]). The metaplastic changes—assessed by upper endoscopy and histological approval—comprise proximal columnar epithelia with intestinal type goblet cells, the junctional (cardial) subtype with mucous secreting glands and the gastric fundus subtype with parietal and chief cells [7, 14]. Up to now, a uniform definition of Barrett's esophagus (BE) remains controversial (e.g., which type of metaplastic changes qualifies the diagnosis BE?) leading to the striking statement “no goblets, no Barrett's” [4, 5], disregarding that nongoblet elements may also be involved in the malignant transformation of BE assessed by Sucrase-Isomaltase and dipeptidyl peptidase IV protein expression [15].

Whereas the detection of intestinal goblet cells in BE samples is already established by using histochemical staining like Alcian-PAS, the diagnosis of dysplasia in BE remains a great challenge due to inter- and intraobserver variation in histology grading (discussed later); therefore, the incidence of dysplasia inside BE varies from 5, to 10% according to national screening efforts and surveillance programs [16]. While diagnostic criteria of BE with dysplasia are relatively well defined by combining cytological and architectural changes, their prospective validation is still missing (for details, see [17–21]).

Moreover, diagnosis of the progression from BE with dysplasia to invasive EAC becomes sometimes impracticable when biopsies are small and criteria of invasiveness are mimicked by distorted rearrangement of glandular structures caused by ulceration and inflammation. At present, using the grade of dysplasia in BE represents the best biomarker in predicting the progression probability for nondysplastic BE (about 0.5%), low-grade dysplasia in BE (13%), and up to 40% in high-grade dysplasia in BE [22, 23]. Therefore, screening surveillance of BE and dysplasia remains still important to detect precursor lesions of EAC in order to avert the disastrous fate of progressive EAC which is characterized by an overall 5-year survival rate between 3.7% and 15.6% [24].

(ii) Complexity factor “diagnosis”: Several issues in BE as well as in EAC detection are still unsolved. The majority of patients with BE remain undiagnosed [25–28], and/or patients with BE and dysplasia are often mis- or overdiagnosed due to inter- and intra-observer errors [10, 29, 30]. Based on the low progression rate of BE to EAC [11], endoscopic and bioptic surveillance studies could not convey a significant benefit for controlled patients [31]. Therefore, the demand for reliable biomarkers regarding prognosis and prediction of patients with BE without/with dysplasia as well as with EAC still remains indispensable. 

(iii) Definition of predictive and prognostic factors (for reviews, see [32–34]): The widely used term “biomarker” represents a marker for physiological or pathological processes or therapeutic response. The clinical characteristics or endpoints (like patient performance status or disease-free period) which should be achieved by these biomarkers as well as methods applied (e.g., genome, transcriptome, proteome, or metabolome) are rather heterogeneous. The term “predictive factor” refers to the use as biomarker for prediction of the statistical probability of disease recurrence, metastasis, or tumor-related death as well as for prediction of specific therapeutic effectiveness.

As recommend by Pepe et al. [35] and McShane et al. [36], different and clearly defined “milestones” must be passed during biomarker development to evaluate their clinical prognostic and predictive potentials: starting with data obtained from experimental cell culture up to retrospective and prospective validation studies resulting in clinical applicability and significant decreasing mortality, and completed by increasing health and cost benefits.

## 3. “Classical” Genetic and Molecular Alterations in GERD, BE, and EAC

During carcinogenesis of BE to EAC, heterogeneous hallmarks of molecular changes are described in the literature [8, 37, 38]. 

(a) Genetic abnormalities of BE include loss of genetic information (especially loss of 9p21, 5q, 13q, 17p, and 18q), whereas for progressive disease, a more extensive imbalance including gain of genetic information (especially gain of 2p, 8q, and 20q) is observed. Finally, enhanced chromosomal instability could be found in the progressive lesions of EAC. 

(b) These genetic abnormalities cause consecutive deregulations of their products like tumor suppressor genes (p53 (loss of 17p), p16 (loss of 9p21), fragile histidine triad protein (FHIT), adenomatous polyposis coli (APC) (loss of 5q), retinoblastoma (Rb) (loss of 13q)), cell cycle regulatory factors (cyclin D1 and MDM2 (mouse double minute 2 homolog)), growth factor receptors (EGFR (epidermal growth factor receptor), TGF-*α* (transforming growth factor)), c-erbB2 and cell adhesion molecules (E- and P-Cadherin and *α*- and *β*-Catenin), as well as proteases (uPA, urokinase-type plasminogen activator) according to the hallmarks of cancer [39]. Additionally, molecular alterations are associated with epigenetic changes such as the methylation and acetylation status as known for APC [40] and p16 [41]. 

(c) Distinct changes in expression pattern of various miRNAs (microRNA) have been demonstrated in BE or EAC. miRNAs are small regulative noncoding RNA molecules (18–22mer) which inhibit the expression of their target genes on posttranscriptional levels; about 30% of human genes are estimated to be regulated by miRNAs [42].

Using global miRNA expression profiling or *in situ* hybridization, several miRNAs (miR-143, -199a_3p, -199a_5p, -100, -99a [43], miR-16-2, -30E, and -200a [44]) have been identified whose expression was associated with reduced overall survival in EAC [43, 44]. A more detailed insight into the relevance of different miRNA expression has been provided recently by Leidner et al. [45] in *n* = 20 EAC samples; next generation sequencing and qRT-PCR identified a total of 26 miRNA that are deregulated in EAC more than 4-fold in >50% of cases compared to normal esophageal squamous tissue. After laser microdissection-based comparison between the steps of BE-EAC-sequence, two miRNAs (miR-31 and -31*) were downregulated in high-grade dysplasia and EAC cases, thus implicating an association with the transition from BE to HGD lesions. Another miRNA (-375) was exclusively down-regulated in EAC, whereas BE and HGD lesions showed normal expression. In a 5-year follow-up study, a different set of miRNAs (miR-192, -194, -196a, and -196b) could be identified in BE samples with progression to EAC compared to patients who did not progress to EAC [46]. The relevance of miRNA-196a as molecular markers associated with the progression from intestinal metaplasia to EAC has also been demonstrated earlier by Luzna et al. [47]. 

Recently, a link between EMT and miRNA expression in BE or EAC was established in both: Barrett's epithelia and EAC displayed a reduced expression of miRNA-200 family members [108]. These miRNAs take a central position in regulation of the initial step of metastasis by inhibiting the EMT effector transcription factors ZEB-1 and -2 [109]. 

Taken together, the relevance of miRNA for prognosis and progression of BE and EAC is being unveiled in current research. Final statements require additional studies using independent patient cohort—also with higher case-load—accompanied by functional verification [43, 110].

## 4. Predictive and Prognostic Factors for GERD, BE, and EAC?

Previous reviews already discussed the importance of biomarkers in this area and proclaimed further investigations thereof in gastroenterological oncology (for review, see Ong et al. [111], Fang et al. [112], and Huang and Hardie [113]). Usually, biomarkers are classified as markers for risk evaluation in patients with GERD to develop EAC or as biomarkers for predictive and prognostic evaluation in patients with diagnosed EAC. Hence, the presented data implicate—and pretend—that we have already reached “the end of the road” with available and significant biomarkers. However, detailed assessment and comparison with other cancers, such as breast, prostate, as well as colorectal [114], reveal them in a rather disillusioning light. Since endoscopic-bioptic surveillance studies yielded no significant benefit for BE patients [31], and prognosis of patients with EAC still is disastrous [24], further intensive experimental and clinical research of (molecular) pathological mechanisms are required urgently. 

**Figure 1:**
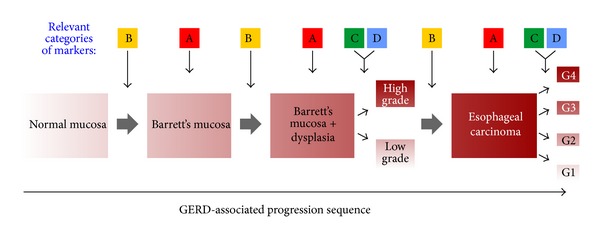
GERD-associated progression for Barrett's esophagus (BE) to esophageal adenocarcinoma (EAC). A–D refer to biomarkers which could be most relevant at the indicated stages of the disease progression (according to Table 1). Therefore A, B, C, and D stand for diagnostic, progressive, predictive, and prognostic biomarkers, respectively.

**Figure 2:**
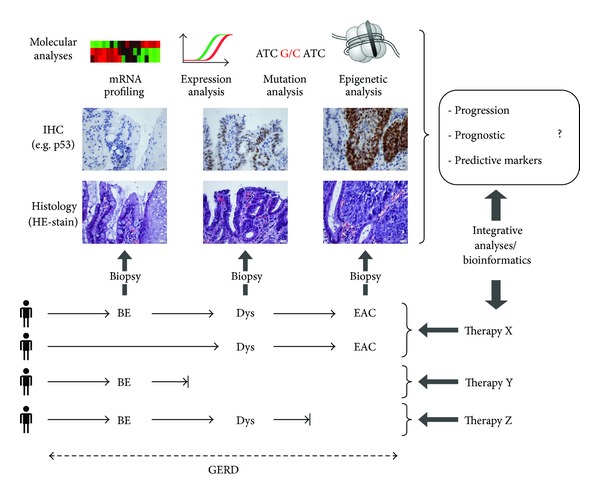
Proposed approach for identification of novel biomarkers for the GERD-BE-AEC sequence. Based on theheterogeneous and patient-specific progression sequence from BE to EAC, the figure indicates the disease stages and mandatory (histology, IHC) and supplementary potential methods for investigation of putative biomarkers for progression, prediction, and prognosis. These data possibly result in an evidence-based stratification of patients for various available therapies (X–Z) based on a rational selection and evaluation of specific biomarkers. Abbreviations. Esophageal adenocarcinoma: AEC; dysplasia: Dys; fluorescence *in-situ* hybridization: FISH; gastro-esophageal reflux disease: GERD; immunohistochemistry: IHC.

Based on studies regarding potential predictive and prognostic markers within the GERD-BE-EAC sequence, we classified them into four groups (Table 1 and Figure 1) and illustrated a patient-specific disease sequence (Figure 2; for details, see reviews [111–113, 115]): (A) diagnostic biomarkers—indicate the presence of disease, (B) progression biomarkers—indicate the risk of developing cancer, that is, progression from BE to EAC, (C) predictive biomarkers—predict response to therapy, and (D) prognostic biomarkers—indicate overall survival, that is, prognosis for EAC.

### 4.1. A = Diagnostic Biomarkers—Indicate the Presence of Disease

The conventional approach for detection and diagnosis is the histochemical analysis of endoscopically derived biopsies of the gastro-esophageal junction, albeit the proposed importance of histological subtypes, the gastric fundus, the cardiac subtype, and the metaplastic columnar epithelium with intestinal-type goblet cells remains unclear [116]. The relevance of these factors has been discussed for years, but prospective studies clarifying the prognostic ability of these histological subtypes are currently not available. Additionally, the trefoil factor 3 (TFF3) combined with a noninvasive diagnostic technique has been investigated intensively in otherwise asymptomatic BE patients [48, 49]. Their results are promising, possibly enabling a selective screening of patients; however, these findings require independent validation and assessment before further clinical application.

### 4.2. B = Progression Biomarkers—Indicate the Risk of Progression from BE to EAC

Similar to the situation for diagnostic biomarkers (A), the most frequently applied progression marker for clinicians and pathologists is the degree of dysplasia in obtained biopsies. Although the inter- and intra-observer error [10, 29, 30] is extremely unsatisfying, studies confirmed that high-grade dysplasia is associated with a 40% higher risk for progression of BE to EAC [22, 23]. Therefore, a primary goal should be the standardization of criteria for dysplasia based on conventional Haematoxylin-Eosin-stained specimens in order to avoid under- and overdiagnosis [10, 29, 30]. Several molecular markers are evaluated too (see Table 1)—the most promising ones according to their statistical robustness (based on OR and RR) are MCM2 expression pattern (highest OR of about 136, whereby the confidence interval is large, reducing the potency of this marker). Loss of heterozygosity on distinct gene loci, especially at 17p, indicates a high progression probability from BE to EAC. The expression pattern of P53 as well as the hypermethylation of p16 and APC suggests high potency, followed by the cell-cycle-associated proteins Cyclin A and D1. These markers were intensively evaluated within retrospective studies but did not succeed the direct transfer to clinical practice, especially due to cost- and time-intensive experimental work. In our experience, the immunohistochemical evaluation of P16 and P53 is well established in pathological diagnostics, whereby the quantification and standardization remains still an unsolved problem.

### 4.3. C = Predictive Biomarkers—Predict Response to Therapy

As displayed in Table 1, the number of potential predictive biomarkers is considerably lower than all other categories accompanied by mainly nonsignificant *P* values. Additionally, biomarkers of category A such as p53 and p16 are also listed in category C, indicating the overall impact of these biomarkers. In sum, the limited number of available and reliable C-markers must be considered as a starting point for inevitable research in the establishment of reliable predictive biomarkers.

### 4.4. D = Prognostic Biomarkers—Indicate Overall Survival–Prognostic in EAC

It is not surprising that the majority of biomarkers are listed in the last category—displaying the typical survey of hallmarks of cancer [39] reaching from self-sufficiency in growth signals (Cyclin D1, EGFR, Ki-67, Her2/neu, TGF-*α*), insensitivity to growth inhibitory signals (TGF-*β*1, APC, P21), evasion of programmed cell death (Bcl-2, COX-2, NF-*κ*B), limitless replicative potential (Telomerase), sustained angiogenesis (CD105, VEGF), invasion and metastasis (Cadherin, uPA, TIMP), tumor differentiation (MGMT), and cancer-related inflammation (NF-*κ*B, COX-2) (see Table 1). Beside their functional heterogeneity, their applicability for prognosis is uncertain. How to use which markers and when? Should we use a panel of markers? The primary and secondary literature currently gives no further advice to solve this problem. Although high levels of significance could be achieved using these biomarkers (*P* < 0.001), the practicability and efficiency in daily routine is unknown. This observation is supplemented by the fact that the most applicable approach for prognostic stratification in EAC is based on the TNM system using conventional basic clinical and pathological findings of tumor extension as well as local and distant metastasis in lymph nodes and organs [117]. Therefore, intensive statistical analysis of comprehensive sets of EAC samples accompanied by selected biomarkers must be performed using factor or hierarchical cluster analysis to evaluate the best prognostic combination of biomarkers. 

To assemble the sometimes confusing data on possible biomarkers (as listed in Table 1) in one point, the histological confirmation of “dysplasia” seems to be unique indicating the “limitation or limited outcome” of our biomarker repertoire (see Table 2). Nevertheless, we should keep in mind that BE is frequently under- and over-diagnosed resulting in huge inter- and intra-observer errors [10, 29, 30], thus demanding for detailed and decisive morphological criteria. From the set of molecular markers, “only” p53, p16, and p21 currently represent applicable biomarkers, especially for progression. Interestingly, growth factors and cell cycle associated factors are relevant for prognosis, but it seems impossible to highlight one exclusively out of the “myriad” of biomarkers [118].

Finally, two major questions arise and are still unsolved: (i) why are proposed biomarkers not (yet) really embedded in clinical routine, and (ii) what impairs the identification of more reliable and significant biomarkers? 

First of all, two major limitations are the technical and financial aspects. Special molecular biological techniques require fresh frozen samples; DNA-, RNA-extraction, and nucleic acid amplification as well as subsequent hybridization or sequencing are time-consuming and need special facilities which are, again, cost intensive. Additionally, validation of specific methods to detect genetic and epigenetic alterations is still not completed. In conclusion, costs and practicability of these biomarkers are the limiting factors until now [111].

Possible answers to the second question are that more relevant entities like inflammation or epithelial-mesenchymal-transition (EMT), which have yet not been completely considered, should be integrated in the evaluation-process of biomarkers for GERD, BE and EAC.

The potential role of the localized inflammation in disease prediction and prognosis is currently rather underestimated in experimental and clinical investigations. Generally, it has been shown that inflammation influences cancerogenesis by key mediators including reactive oxygen species (ROS), NF-*κ*B, inflammatory cytokines, prostaglandins, and specific microRNAs (miRNAs) [119]. Poehlmann et al. comprehensively reviewed the role of inflammation on genetic and epigenetic changes in BE and EAC focusing on oxidative stress and the NF-*κ*B-pathway [120]. Beside NF-*κ*B and COX-2 (see Table 1), other transmitters of inflammation like chemokines or cytokines should be investigated as possible biomarkers. 

Additionally, the process of EMT with its key players Snail, Twist, and ZEB and their repressed target protein E-Cadherin is essentially linked to development, regeneration, inflammation, and cancerogenesis [121]. Several ontogenic pathways (e.g., WNT-, Hedgehog-, or Notch-signaling) are involved in EMT regulation and have also been associated with pathogenesis of BE to EAC as reviewed by Chen et al. [38]. Furthermore, increased expression of SLUG is associated with progression of EAC by consecutive repression of E-Cadherin indicating a role of EMT in EAC. Therefore, subsequent clinical trials have to be set up to elucidate distinct mechanisms of EMT in the pathogenesis of or as specific biomarkers in BE or EAC [122].

## 5. Approach and Outlook

The probability to find one single specific biomarker providing all diagnostic, predictive, and prognostic significance in GERD, BE, and/or EAC is rather utopian, and a panel of biomarkers maybe will solve this problem [81, 123, 124]. Upcoming new technologies such as RNA and DNA microarrays, epigenetics, and proteomics in association with bioinformatics give hope to find novel and reliable biomarkers in gastrointestinal tumors and especially for prognosis and prediction of BE and EAC [114]. These technologies may provide insights in this rather complex sequence of GERD-BE-EAC; for instance, Kaz et al. [125] stratified BE and EAC by methylation signatures and molecular subclasses using DNA methylation profiling. Interestingly, the authors found an increase of methylation during disease progression—supporting the postulated GERD-BE-EAC sequence and promoting studies of biomarkers based on epigenetic mechanisms which are specific for particular steps in the pathogenetic sequence. Additionally, miRNA profiling by Ko et al. [126] discovered five miRNAs which are significantly expressed in patients with EAC with and without complete remission after therapeutic interventions, whereby the connection of these interesting data to other prognostic/predictive biomarkers in EAC has not been performed. As mentioned by Jankowski and Odze [114], the new technologies are associated with “specific” limitations; RNA and DNA array techniques are retrospective and are frequently lacking phenotype controls. Epigenetic experimental approaches often showed an overlap of methylation pattern between normal and precancerous tissues with no possibility of discrimination between them. Proteomics is time-consuming and not applicable for daily routine work. This seems also true for bioinformatics' techniques.

As depicted in Figure 2, every stage of disease demands intensive morphological, genetic, as well as epigenetic analysis and consequently an exorbitant research effort due to the heterogeneity within the GERD-BE-EAC sequence. However, only consistent generating of data from patients with GERD, GERD with BE, GERD with EAC, or GERD with BE and EAC will allow integrative analysis and research, even if this implies that patients with GERD will be under consecutive, perhaps lifelong surveillance. Therefore, consolidation and evaluation of our intensive but partial not coherent findings regarding the “puzzle” of GERD-BE-EAC represent the first steps to discover the best biomarkers for diagnosis, therapy, and prognosis.

## Figures and Tables

**Table 1:** Summary of investigated and published biomarkers in the GERD-BE-EAC axis. The categorization is based on four groups according to their potential usage as A = Diagnostic Biomarker indicates the presence of disease, B = Progression Biomarker indicates the risk of developing cancer—progression in BE to EAC, C = Predictive Biomarker predicts response to therapy (CTX, RTX, photodynamic therapy), or D = Prognostic Biomarker indicates overall survival—prognostic in EAC (survival, recurrence).

	Biomarker	Method	Remarks/findings	OR/RR/*P* value	Refs
A = Diagnostic Biomarker	TFF3		novel nonendoscopic screening modality in a prospective cohort study	*P* = 0.02 (for maximal length of BE) *P* = 0.009 (for circumferential length of BE)	[48]
	TFF3	IHC, esophageal cytosponge samples for BE combined with IHC for TFF3	biomarker to screen asymptomatic patients for BE; TFF3 protein was expressed at the luminal surface of BE (not at normal esophageal or gastric mucosa)	*P* < 0.0001	[49]
	Chromosomes 7 and 17 (copy number changes)	ICDA & FISH	chromosomal gains in early stages of BE; valuable adjunct to conventional cytology to detect dysplasia or EAC	IND/LGD: 75% sensitivity, (76% specificity) HGD/EAC: 85% sensitivity, (84% specificity)	[50]
	8q24 (*C-MYC*), 17q12 (*HER2*), and 20q13 (copy number changes)	FISH	chromosomal gains in early stages of BE; represents a valuable adjunct to conventional cytology to detect dysplasia or EAC	LGD (50% sensitivity) HGD (82% sensitivity) EAC (100% sensitivity)	[51]
	17q11.2 (*ERBB2*)	Southern blotting, microarray analysis	amplified copies of the *ERBB2* gene in EAC	10-fold amplification in 3 of 25 (12%) tumors	[52]
	Serum proteomic pattern analysis	mass spectrometry	several limitations due to applied technology	identified 10 of 11 normal's; and 42 of 43 EAC's correctly	[53]

B = Progression Biomarkers	P53 positivity	IHC	limited efficacy as a single progression biomarker	OR 11.7 (95% CI: 1.93–71.4)	[54]
	P53 positivity	IHC	positive in 4/31 that regressed, 3/12 that persisted, and 3/5 that progressed to HGD or EAC	RR not available	[55]
	DNA content abnormalities	flow cytometry	higher relative risk for EAC in patients with tetraploidy (4N) or aneuploidy (>6%)	tetraploidy: RR 7.5 (95% CI: 4–14) (*P* < 0.001) aneuploidy: RR 5.0 (95% CI: 2.7–9.4) (*P* < 0.001)	[56]
			4N fraction cut point of 6% for cancer risk	RR 11.7 ( 95% CI: 6.2–22)	
			aneuploid DNA contents of 2.7N were predictive of higher cancer risk	RR 9.5 (95% CI: 4.9–18)	
	DNA content abnormalities	flow cytometry	presence of both 4N fraction of 6% and aneuploid DNA content of 2.7N is highly predictive for progression	RR 23 (95% CI: 10–50)	[57]
			17p(p53) LOH associated with higher risk of progression to HGD + EAC	HGD: RR 3.6 (*P* = 0.02)	
		flow cytometry, PCR		EAC: RR 16 (*P* < 0.001)	[58]
			combined LOH of 17p and 9p and DNA content abnormalities can best predict progression to EAC	RR 38.7 (95% CI: 10.8–138.5) not clinical applicable	
	LOH of 157p and 9p and DNA content abnormalities		LOH of 17p alone	RR 10.6 (95% CI: 5.2–21.3)	
		flow cytometry, PCR	LOH of 9p alone	RR 2.6 (95% CI: 1.1–6.0)	
			Aneuploidy alone	RR 8.5 (95% CI: 4.3–17.0)	[59]
			Tetraploidy alone	RR 8.8 (95% CI: 4.3–17.7)	
	mutations of *p16* and *p53 *loci (clonal diversity measurements)	flow cytometry, PCR	significant predictors for EAC progression, not clinical applicable	*P* = 0.001	[60]
	EGFR	IHC	overexpression in HGD/EAC	35% of HGD/80% of EAC specimens	[61]
	MCM2	IHC	correlation between degree of dysplasia and level of ectopic luminal surface MCM2 expression	MCM2-positive staining in 42% (19/45) of BE samples	[62]
	Cyclin A	IHC	surface expression of cyclin A in BE samples correlates with the degree of dysplasia	OR 7.5 (95% CI: 1.8–30.7) (*P* = 0.016)	[63]
	Cyclin D1	IHC	association with increased risk of EAC	OR 6.85 (95% CI: 1.57–29.91)	[64]
	hypermethylation of *p16* (CDKI2A)		association with increased risk of progression to HGD/EAC	OR 1.74 (95% CI: 1.33–2.2)	
	hypermethylation of *RUNX3 *		association with increased risk of progression to HGD/EAC	OR 1.80 (95% CI: 1.08–2.81)	
	hypermethylation of HPP1	RT-PCR	association with increased risk of progression to HGD/EAC	OR 1.77 (95% CI: 1.06–2.81)	[41]
	hypermethylation of *p16* and APC	PCR	predictor of progression to HGD/EAC	OR 14.97 (95% CI: 1.73–inf.)	[65]
	8 gene methylation panel	RT-PCR	age dependent; predicts 60.7% of progression to HGD/EAC within 2 yrs	RR not available (90% specificity)	[66]
	Gene expression profile	microarray analysis	64 genes up regulated 110 genes down regulated in EAC	*P* = 0.05	[67]
	Cathepsin D, AKR1B10, and AKR1C2 mRNA levels	Western blotting, qRT-PCR	dysregulation predicts progression to HGD/EAC	AKR1C2: ↑ levels in BE (*P* < 0.05) but ↓ levels in EA (*P* < 0.05)	[68]
		ICDA	aneuploidy predicts progression to EAC	60% with LGD; 73% with HGD, and 100% with EAC (total number of samples = 56)	[69]
	DNA abnormalities	ACIS	frequency and severity of aneuploidy predicts progression to EAC	unstable aneuploidy in 95% with EAC	[70]
		DICM	relationship between DICM status and progression to HGD/EAC	*P* < 0.0001	[71]
	SNP-based genotyping in BE/EAC specimens	flow cytometry, 33K SNP array	copy gains, losses, and LOH increased in frequency and size between early and late stage of disease	*P* < 0.001 (BE)	[72]

C = Predictive Biomarkers	*p16* allelic loss	FISH	decreased response to photodynamic therapy	OR 0.32 (95% CI: 0.10–0.96)	[73]
	DNA ploidy abnormalities	ICDA	DNA ploidy as a covariate value for recurrence	HR 6.3 (1.7–23.4) (*P* < 0.0015)	[74]
	HSP27	IHC	association between low HSP27 expression and no response to neoadjuvante chemotherapy	*P* = 0.049 and *P* = 0.032	[75]
	Ephrin B3 receptor	microarray	response prediction in EAC in patients with Ephrin B3 receptor positive versus Ephrin B3 receptor negative	Response rate <50%: 3 (15.8) versus 16 (84.2) (*P* < 0.001)	[76]
	Genetic polymorphisms	qRT-PCR	association between individual single nucleotide polymorphisms and clinical outcomes	comprehensive panel of genetic polymorphisms on clinical outcomes in 210 esophageal cancer patients	[77]
	P21	IHC	alteration in expression correlated with better CTX-response	*P* = 0.011	[78]
	P53	IHC	alteration in expression correlated with better CTX-response	*P* = 0.011	[79]
	ERCC1	IHC	ERCC1-positivity predicts CTX-resistance and poor outcome	*P* < 0.001	[80]

D = Prognostic Biomarkers	DCK PAPSS2 SIRT2 TRIM44	RT-PCR, IHC	prognostic 4-gene signature in EAC predicts 5-year survival	0/4 genes dysregulated: 58% (95% CI: 36%–80%) 1-2/4 genes dysregulated: 26% (95% CI: 20%–32%) 3-4/4 genes dysregulated: 14% (95% CI: 4%–24%) (*P* = 0.001)	[81]
	*p16* loss *C-MYC *gain	FISH	association between therapy response status and FISH positivity	*P* = 0.04	[82]
	ASS expression	microarrays	low expression correlates with lymph node metastasis	*P* = 0.048	[83]
	microRNA expression profiles	miRNA microarray, qRT-PCR	association with prognosis (e.g. low levels of mir-375 in EAC → worse prognosis)	HR = 0.31 (95% CI: 0.15–0.67) (*P* < 0.005)	[84]
	Genomic alterations	MLPA	reverse association between survival and DNA copy number alterations (>12 aberrations → low mean survival)	*P* = 0.003	[85]
	Cyclin D1	FISH, IHC	2 of 3 genotypes confers to ↓ survival	*P* = 0.0003	[86]
		IHC	expression = ↓ survival	*P* = 0.07	[87]
	EGFR	IHC	↓ expression = ↓ survival	*P* = 0.034	[88]
	Ki-67	IHC	low levels of staining (<10%) = ↓ survival	*P* = 0.02	[89]
	Her2/neu	FISH	amplification = ↓ survival	*P* = 0.03	[90, 91]
		IHC	low levels = ↓ survival	*P* = 0.03	[92]
	TGF-*α*	IHC, ISH	high levels = tumor progression and lymph node metastasis	*P* = 0.025 and *P* < 0.05	[93]
		qRT-PCR	overexpression = ↓ survival	*P* = 0.0255	[94]
	TGF-*β*1	ELISA	high plasma levels = ↓ survival	*P* = 0.0317	[95]
	APC	RT-PCR	high plasma levels of methylation = ↓ survival	*P* = 0.016	[96]
	Bcl-2	IHC	expression = ↓ survival	*P* = 0.03	[97]
		IHC, RT-PCR	↑ expression = ↓ survival, ↑ TN-stage, and recurrence	*P* < 0.001, *P* = 0.008/0.049, and *P* = 0.01	[98]
		IHC	strong staining = ↓ survival	*P* = 0.03	[99]
	COX-2	IHC	strong staining = ↓ survival, distant metastasis, and recurrence	*P* = 0.002, *P* = 0.02, and *P* = 0.05	[100]
	NF-*κ*B	IHC	activated NF-*κ*B = ↓ survival, and ↓ disease free survival	*P* = 0.015 and *P* = 0.010	[101]
	Telomerase	Southern blot analysis, RT-PCR	higher telomere-length ratio = ↓ survival	RR of death: 3.4 (CI: 1.3–8.9) (*P* < 0.02)	[102]
			expression = ↓ survival,	*P* < 0.01	
	CD105		angiolymphatic invasion	*P* < 0.05	
			↑ lymph node metastasis	*P* < 0.01	
			↑ T-stage	*P* < 0.001	
		IHC	↑ distant metastasis	*P* < 0.01	[103]
			↑ expression = ↓ survival,	*P* < 0.01	
	VEGF		angiolymphatic invasion	*P* < 0.05	
			↑ lymph node metastasis	*P* < 0.01	
			↑ T-stage	*P* < 0.01	
			↑ distant metastasis	*P* < 0.01	
	Cadherin	IHC	↓ level = ↓ survival	*P* = 0.05	[89]
	uPA	ELISA	↑ uPA = ↓ survival	*P* = 0.0002	[104]
	TIMP	IHC, RT-PCR	↓ expression = ↓ survival, and ↑ disease stage	*P* = 0.007 and *P* = 0.046	[105]
	Promoter hypermethylation of multiple genes	IHC, methylation specific PCR	if >50% of gene profile methylated = ↓ survival, and earlier recurrence	*P* = 0.05 and *P* = 0.04	[106]
	MGMT hypermethylation	IHC, methylation specific PCR	correlation with higher tumor differentiation	*P* = 0.0079	[107]

ACIS: automated cellular imaging system; ASS: argininosuccinate synthase; APC: adenomatous polyposis coli; BE: barrett's esophagus; COX: cyclooxygenase; DCK: deoxycytidine kinase; DICM: digital image cytometry; EAC: esophageal adenocarcinoma; EGFR: epidermal growth factor receptor; ELISA: enzyme-linked immunosorbent assay; FISH: fluorescence *in-situ*-hybridization; ICDA: image cytometric DNA analysis; HSP27: Heat-shock protein 27; IHC: immunohistochemistry; LOH: loss of heterozygosity; PAPSS2: 3′-phosphoadenosine 5′-phosphosulfate synthase 2; PCR: polymerase chain reaction; qRT: quantitative reverse transcriptase; MLPA: multiplex ligation dependent probe amplification; NF-*κ*B: nuclear factor kappa B; SIRT2: Sirtuin 2; SNP: single nucleotide polymorphism; TFF3: Trefoil factor 3; TGF: transforming growth factor; TIMP: tissue inhibitors of metalloproteinases; TRIM44: Tripartite motif-containing 44; uPA: urokinase-type plasminogen activator; VEGF: vascular endothelial growth factor.

**Table 2:** Synopsis of biomarkers in the GERD-BE-EAC axis. According to Table 1, most promising biomarkers are summarized indicating that only dysplasia is involved in all four categories. Dysplasia can be used as diagnostic biomarker as well as to assess the risk of progression to EAC or response to therapy and is associated with poor survival (↓ survival).

	Dysplasia	P53	P16	P21	Growth factors	Cell cycle
A = Diagnostic Biomarker	*✓*					
B = Progression Biomarker	*✓*	*✓*	*✓*	*✓*	*✓*	*✓*
C = Predictive Biomarker	*✓*	*✓*	*✓*	*✓*		
D = Prognostic Biomarker	↓ survival		*✓*		↓ survival	↓ survival

## Abbreviations

ACIS: Automated cellular imaging system
ASS: Argininosuccinate synthase
APC: Adenomatous polyposis coli
BE: Barret's esophagus
COX: Cyclooxygenase
DCK: Deoxycytidine kinase
DICM: Digital image cytometry
EAC: Esophageal adenocarcinoma
EGFR: Epidermal growth factor receptor
ELISA: Enzyme linked immunosorbent assay
EMT: Epithelial-mesenchymal-transition
FHIT: Fragile histidine triad protein
FISH: Fluorescence *in-situ*-hybridization
GERD: Gastro-esophageal reflux disease
ICDA: Image cytometric DNA analysis
HSP27: Heat-shock protein 27
IHC: Immunohistochemistry
LOH: Loss of heterozygosity
PAPSS2: 3′-phosphoadenosine 5′-phosphosulfate synthase 2
PCR: Polymerase chain reaction
qRT: Quantitative reverse transcriptase
MDM2: Mouse double minute 2 homolog
MLPA: Multiplex ligation dependent probe amplification
NF-*κ*B: Nuclear factor kappa B
Rb: Retinoblastoma
SIRT2: Sirtuin 2
SNP: Single nucleotide polymorphism
TFF3: Trefoil factor 3,
TGF: Transforming growth factor
TIMP: Tissue inhibitors of metalloproteinases
TRIM44: Tripartite motif-containing 44
uPA: Urokinase-type plasminogen activator
VEGF: Vascular endothelial growth factor.
